# Patient Adherence to Glaucoma Treatment During the COVID-19 Pandemic

**DOI:** 10.7759/cureus.15545

**Published:** 2021-06-09

**Authors:** Ioanna Mylona, Maria Dermenoudi, Nikolaos M Glynatsis, Mikes N Glynatsis

**Affiliations:** 1 Ophthalmology, General Hospital of Katerini, Katerini, GRC; 2 Ophthalmology, Health Center of Neapolis, Thessaloniki, GRC; 3 Ophthalmology, Medical University of Plovdiv, Plovdiv, BGR; 4 Ophthalmology, ‘Hippokration’ General Hospital of Thessaloniki, Thessaloniki, GRC

**Keywords:** covid-19, glaucoma, arms2, medication adherence strategies, patient adherence

## Abstract

Introduction

In medicine, patient adherence refers to the degree to which a patient correctly follows medical advice, and it most commonly pertains to medication or drug compliance. Patient adherence to glaucoma treatment has been a frequent and serious issue that is associated with adverse long-term outcomes. The purpose of this study was to determine the factors that are associated with adherence to glaucoma treatment among patients during the ongoing coronavirus disease 2019 (COVID-19) pandemic.

Methods

This was a cross-sectional study involving 100 consecutive glaucoma outpatients who were interviewed based on the modified version (ARMS2-COVID) of the original Adherence to Refills and Medications Scale 2 (ARMS2) that examined adherence to medication. Length of treatment and disease onset along with basic demographic details (gender, age, socioeconomic status, and educational levels) of the patients were also recorded.

Results

The COVID-19 pandemic has disproportionally impacted patients of older age (p=0.033) and lower educational levels (p<0.001) with regard to their ability to follow their treatment plan regardless of the duration of previous treatment.

Conclusions

Based on our findings, in order to ensure higher levels of patient adherence among patients of older age with limited education, more planning will be required, aided by appropriate educational interventions and proactive patient follow-ups.

## Introduction

Glaucoma was the fourth leading cause of moderate or severe vision impairment globally in 2015, accounting for four million patients, while it was the third leading cause of blindness with 2.9 million affected patients [1]. Glaucoma is a chronic disease, and long-term adherence to medication is vital for its prognosis. However, adherence to medication has been an issue throughout the world; a recent study in South Korea [2] found 27.4% of patients being non-adherent to medication while a related study in Iran [3] found only 34.6% of patients being adherent. The vast difference in findings between the two studies could be attributed to the differences in participants' level of awareness on the need for a stable treatment plan, as well as lower mean educational levels and lower quality of life among Iranian patients. Lack of adherence to glaucoma treatment is associated with progressive visual field loss, with non-adherent patients presenting 80% worse visual field defects compared to adherent ones [4]; moreover, non-adherence could also lead to additional prescriptions or surgery that could be otherwise avoided [5].

In light of the considerable prevailing rate of non-adherence, the issue has been targeted with comprehensive programs that include patient education, motivational tools, and counseling for behavioral changes [6]. However, due to the outbreak and rapid spread of the coronavirus disease 2019 (COVID-19) pandemic, these programs either needed to be in place beforehand to have delivered any significant impact or would be halted due to quarantine measures and restrictions on movement necessitated by the pandemic. Since elderly patients are at higher risk of infection from COVID-19, they have been disproportionally affected by the restrictive measures in place and their outdoor movement has been generally discouraged throughout the world. During the first quarantine period in Greece (March 23-May 4, 2020), outpatient services and scheduled surgeries in ophthalmologic departments were halted and only acute cases were treated in the emergency departments.

## Materials and methods

The inclusion criteria were as follows: patients with a diagnosis of any type of glaucoma, who had been followed up as an outpatient with at least one previous appointment before the pandemic, who were not under treatment for a separate eye disease, and the ability to provide informed consent and fill in the research material independently. A total of 100 consecutive glaucoma outpatients attending the Department of Ophthalmology confirmed their participation in this study, out of the 112 that were initially approached; the reason for non-enrollment was the inability to read the research material independently due to eye disease. The study was carried out during the first outpatient visit that the patients managed to attend after the outpatient services were resumed following the easing of quarantine protective measures.

Patient adherence was evaluated with a Greek-translated, modified version (ARMS-C) of the original Adherence to Refills and Medications Scale 2 (ARMS2) by Kripalani et al. [7], which examined adherence to medication in terms of two factors: (1) correct form and refill of medication and (2) correct intake of medication. The translation process included a forward translation from English to Greek as well as a backward translation. These translations were forwarded to a local translation service for a review of grammar and the correct use of Greek and English. The linguistic validation was run under the supervision of the principal author who tested the draft among 20 patients for appropriateness and comprehension. No change in content was necessary. The final validation of the draft was confirmed by all authors by a unanimous decision.

The original version included a subscale of four items relating to prescription-refill and a subscale of eight items relating to medication-taking; each item was to be answered based on a four-point Likert scale, with the responses ranging from “never,” to “sometimes,” “mostly,” and “always”. In this version, all items were modified to include a clear reference to the time frame of the COVID-19 pandemic in contrast to the usual practice. Three new items were added to the original 12-item version: an item that queried on missed appointments due to the COVID-19 pandemic, an item that queried whether the patient was out of medication and had to seek them from another patient, and an item that enquired whether the patient managed to contact his/her ophthalmologist during the pandemic. The ARMS-C questionnaire is presented in the Appendix section. As a criterion of reliability, we assessed Cronbach’s alpha value, which was high at 0.924. Additionally, the patients provided their basic demographic and clinical information, including gender, age, living arrangements, educational level, duration of glaucoma diagnosis, type of medication, and frequency of scheduled appointments before the pandemic. Living arrangements were presumed to be relevant with regard to elderly patients since they relate to possible supervision on medication adherence.

Among the patients, 48 were male (mean age=70.1 years, SD=10.4 years) and 52 were female (mean age=68.35 years, SD=9.32 years) Of note, 48% of patients lived alone, 38% with their spouse or partner, and 7% with their children; 32% of the patients had completed high school or higher education. The mean duration since the glaucoma diagnosis was 7.58 years (SD=7.1 years), while the mean duration between follow-up appointments was 6.1 weeks (SD=3.3 weeks).

## Results

The score was not distributed normally and subsequent computations were carried out with non-parametric statistics. The total mean score was 28.72 (SD=9.44) and the scores of the two genders were comparable (Student’s t-test=1.177, p=0.242). Correlational statistics (Table 1) indicated that the ARMS-C total score correlated with age (r_s_=0.51, p<0.001) and years of diagnosis (r_s_=0.324, p=0.022). The correlation of ARMS-C total score with age remained significant after controlling for years of diagnosis (r_s_=0.418, p<0.001).

**Table 1:** Correlations of ARMS-C total score *Correlation is significant at the 0.01 level (two-tailed). **Correlation is significant at the 0.05 level (two-tailed) ARMS: Adherence to Refills and Medications Scale

		Age	Years of diagnosis	Regularity of follow-up
ARMS total score	Spearman's r	0.510^*^	0.324^**^	0.187
	P-value	<0.001	0.022	0.193
Years of diagnosis	Spearman's r	0.726^*^	-	-0.154
	P-value	<0.001	.	0.286
Regularity of follow-up	Spearman's r	-0.172	-0.154	-
	P-value	0.232	0.286	.

A multiple regression analysis that included gender, age, years of education, and years of diagnosis led to a statistically significant result, F (4, 99)=17.795, p<0.001, r^2^=0.404 (Table 2), with a higher level of education and a younger age being significant predictors of higher ARMS-C score, while the other two variables did not impact the ARMS-C score. The effect size r^2^ denotes moderate clinical significance for the findings.

**Table 2:** Regression analysis for total ARMS-C score t: t-test value; p: statistical significance value ARMS: Adherence to Refills and Medications Scale

	Standardized beta	t	p	95% confidence interval for beta	
				Lower bound	Upper bound
(Constant)		0.994	0.325	-11.295	33.349
Gender	-0.053	-0.673	0.502	-3,938	1,943
Age	0.363	2.196	0.033	0.029	0.664
Education	-0.420	-4.707	<0.001	-5,278	-2,175
Years of diagnosis	-0.039	-0.365	0.716	-0.332	0.229

## Discussion

Our results indicated that patient adherence (represented by the ARMS-C total score) was lower in patients of older age even after controlling for years since receiving a diagnosis. Another significant demographic was lower educational level. Thus, we may ascertain that there is a significant issue to be addressed during the ever-expanding time frame of the current COVID-19 pandemic: The pandemic has disproportionally affected patients of older age and lower educational levels with regard to their ability to follow their treatment plan, regardless of how much time has elapsed since the diagnosis. A separate finding was that gender was not a factor in patient adherence to treatment.

Our results are in line with a number of other recent studies. A study involving 116 patients in the US identified age as one of the three factors associated with treatment adherence, the other two being the total number of other eye diseases and race [8]. Low self-efficacy and forgetfulness were among the barriers associated with poor adherence in a study of 190 adults taking glaucoma medication in the US [9], these two factors being typically impacted by older age. Lower education was also associated with poor medication adherence in glaucoma patients in a recent literature review [10].

Limitations of this study include its cross-sectional design, and the fact that neither specific treatments nor the degree of glaucoma severity were included in the analysis. We were unable to factor in glaucoma severity due to the requirement in the inclusion criteria that patients should be able to read through and fill in the research material on their own.

More planning will be required to resolve the issues related to patient adherence among older patients and those with low educational levels, with appropriate educational interventions and proactive patient follow-ups; reminder services via text messaging could serve as reminders to improve medication adherence and foster a sense of interest and connectedness to the caregiver; moreover, patients with low educational levels will not find these methods challenging as these are easy to interpret and use. This approach has been tested successfully for some years now [11], and results have indicated negligible difference with reminders over the telephone and considerably less burden on the staff, with the added advantage of the associated technology being cheap and widely accessible. In a recent study of 989 patients on their views regarding the potential use of automated reminders, 68.9% of the participants reported that text messaging would help them remember appointments, and 47.5% stated that it would help with medications, compared to 40.8% and 18.7% respectively who felt the same about e-mail reminders [12]. Provision of follow-up and monitoring services via the internet may not take into account the difficulty associated with internet use for those with eyesight and educational handicaps and a lack of technical expertise [13], while general knowledge among older patients with low education levels regarding telemedicine is poorer compared to younger ones [14].

## Conclusions

While the direct impact of COVID-19 has been felt by those who have contracted the disease and its sequelae, the pandemic has also indirectly affected a large number of patients who have had their access to healthcare curtailed and scheduled follow-ups canceled or reduced in frequency due to the heavy burden that the pandemic has placed on the healthcare systems globally. In the case of glaucoma follow-up appointments, we have found that older patients with lower educational levels have been disproportionally impacted. Since the pandemic and its consequences will continue to affect us for the foreseeable future, more planning will be required to ensure medication and treatment adherence among older patients with limited education, and this warrants appropriate educational interventions and proactive patient follow-ups.

## Appendices

Table 3 presents the ARMS-C questionnaire.

**Table 3:** ARMS-C questionnaire ARMS: Adherence to Refills and Medications Scale; COVID-19: coronavirus disease 2019

Questionnaire	
1	Did you miss scheduled appointments due to the COVID-19 pandemic?
2	How often did you forget to take your medicine during the COVID-19 pandemic?
3	How often did you decide not to take your medicine during the COVID-19 pandemic?
4	How often did you forget to get prescriptions filled during the COVID-19 pandemic?
5	How often did you run out of medicine during the COVID-19 pandemic?
6	How often did you skip a dose of your medicine during the COVID-19 pandemic?
7	How often did you miss taking your medicine when you felt better during the COVID-19 pandemic?
8	How often did you miss taking your medicine when you felt sick during the COVID-19 pandemic?
9	How often did you take someone else’s medicine during the COVID-19 pandemic?
10	How often did you miss taking your medicine when you were careless during the COVID-19 pandemic?
11	How often did you change the dose of your medicines to suit your needs (like when you took more or less pills than you were supposed to) during the COVID-19 pandemic?
12	How often did you forget to take your medicine when you were supposed to take it more than once a day during the COVID-19 pandemic?
13	How often did you put off refilling your medicines because they cost too much money during the COVID-19 pandemic?
14	How often did you plan ahead and refill your medicines before they ran out during the COVID-19 pandemic?
15	Did you manage to contact your attending ophthalmologist during the COVID-19 pandemic?
